# A Rare Case of Acute Calculous Cholecystitis With Gallbladder Hydrops

**DOI:** 10.7759/cureus.22230

**Published:** 2022-02-15

**Authors:** Nagapratap Ganta, Dina Alnabwani, Viraj Shah, Ayesha Imtiaz, Veera Jayasree Latha Bommu, Pramil Cheriyath

**Affiliations:** 1 Internal Medicine, Hackensack Meridian Ocean Medical Center, Brick, USA; 2 Internal Medicine, Rajarshee Chhatrapati Shahu Maharaj Government Medical College, Kolhapur, IND

**Keywords:** mrcp, cholecystectomy, cholelithiasis, gallbladder mucocele, acalculous gallbladder, acute cholecystitis, gallbladder hydrops

## Abstract

Gallbladder hydrops or mucocele is usually due to the obstruction of the gallbladder by a gallstone. It is usually characterized by an increase in gallbladder volume, which remains clinically silent and is often incidentally diagnosed during exploratory laparotomy or laparoscopy. We report a rare case of acute calculous cholecystitis with gallbladder hydrops (measuring 17 cm in maximum dimension) due to the obstruction of the cystic duct by a gallstone in a 67-year-old female. We highlight the importance of early magnetic resonance imaging (MRI) in patients with right upper quadrant (RUQ) pain to rule out gallbladder hydrops especially in those with a history of gallstones.

## Introduction

Acute calculous and acalculous gallbladder diseases are two types of gallbladder illness; gallbladder hydrops can be either due to calculous or acalculous cholecystitis. Gallbladder hydrops is characterized by an increase in gallbladder volume without any inflammatory symptoms, bacterial infection, or abnormalities of the biliary ducts or gallbladder [1]. One of the features of a positive prognosis is the absence of inflammation, which distinguishes gallbladder hydrops from acute acalculous cholecystitis [2]. Mucocele of the gallbladder is a condition that occurs when the cystic duct is blocked for an extended period, usually due to an impacted gallstone. It is frequently undetected before surgery and is discovered as an afterthought during laparoscopic or open cholecystectomy. When the gallbladder is surgically decompressed and clear mucus-like fluid replaces the green or brown bile, this diagnosis is made. Acute or chronic cholecystitis symptoms are found in patients [3]. In our case report, we describe a case that presented with right upper quadrant (RUQ) pain with suspected acute cholecystitis that was later diagnosed as gallbladder hydrops after diagnostic imaging.

## Case presentation

A 67-year-old female was presented to the emergency department (ED) with complaints of constant upper right and mid abdominal pain, nausea, and vomiting for the past four days. The patient stated that her upper abdominal pain started gradually, radiating to the back, and that it was worse on her right side and was associated with nausea and vomiting. The patient was not COVID vaccinated. Her vitals in the ED were as follows: blood pressure of 151/91 mm Hg, heart rate of 82 beats per minute, respiratory rate of 18 breaths per minute, temperature of 98.6°F, 97% SpO_2_, and body mass index (BMI) of 27.03 kg/m². On examination, the abdomen was soft, moderate abdominal tenderness was elicited in the right upper quadrant (RUQ) and epigastric area, and there was no guarding or rebound, with bilateral inguinal scars that are both well healing with no evidence of recurrence of the hernias.

She has a history of gallstones that was treated conservatively, deep vein thrombosis (DVT) in the right leg, hypothyroidism, bilateral inguinal hernia repairs done in July 2021, appendectomy, tonsillectomy, thyroidectomy, and surgery for varicose veins in the left leg. Her medications included levothyroxine 100 mcg and oxycodone-acetaminophen 5-325 mg every four hours as needed. She denied tobacco, alcohol, and drug use.

She was diagnosed with acute cholecystitis. Intravenous fluids, morphine, ondansetron 4 mg, and piperacillin-tazobactam 3.375 g were administered in the ED. Her complete blood count (CBC) showed elevated white blood cells (WBCs) at 18.8 × 10^3^/uL (normal range: 4.5-11.0 × 10^3^/uL). Her basic metabolic panel, liver function tests, serum electrolytes, and coagulation studies were normal. Urinalysis showed moderate blood with 3-5 cells/HPF.

**Figure 1:**
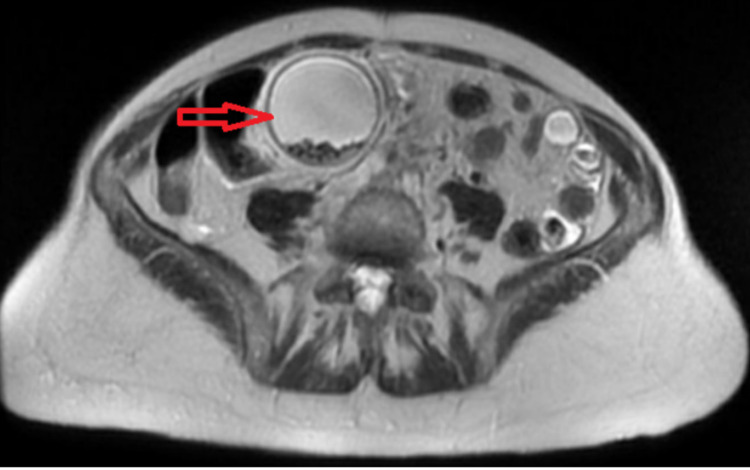
MRI of the abdomen showing the gallbladder hydrops (arrow) MRI: magnetic resonance imaging

**Figure 2:**
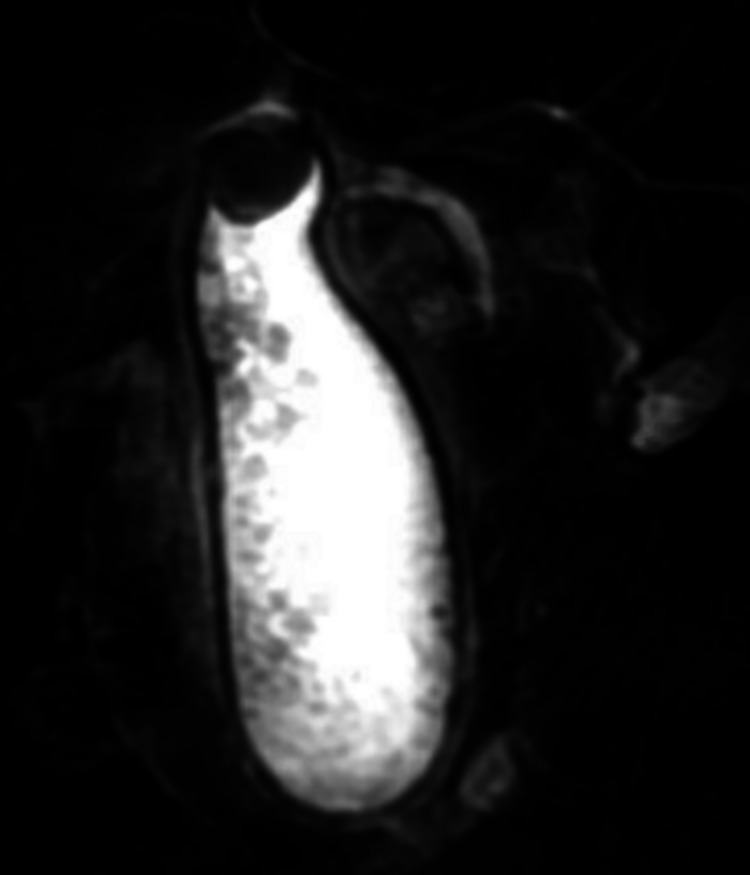
MRCP showing acute calculous cholecystitis with gallbladder hydrops measuring 17 cm in maximum dimension MRCP: magnetic resonance cholangiopancreatography

Ultrasonography (USG) of the RUQ showed markedly dilated gallbladder with a large gallstone in the gallbladder neck with additional smaller stones, wall thickening of 5 mm, and common bile duct (CBD) dilatation of 9 mm. The findings were suggestive of acute cholecystitis. Magnetic resonance imaging (MRI) (Figure 1) and magnetic resonance cholangiopancreatography (MRCP) revealed acute calculous cholecystitis with gallbladder hydrops measuring 17 cm in maximum dimension (Figure 2) with the largest gallstone measuring 2.8 cm.

**Figure 3:**
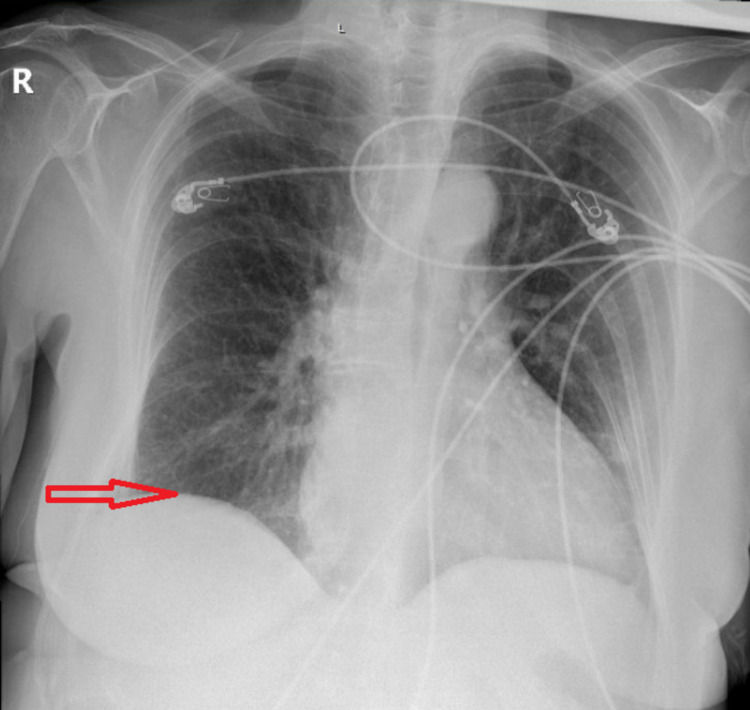
Portable chest X-ray anteroposterior (AP) view showing elevation of the right hemidiaphragm (arrow)

Gallbladder wall thickening and pericholecystic fluid were noted. Distal CBD calculus measuring 4 mm was suspected. A portable X-ray of the chest showed elevation of the right hemidiaphragm (Figure 3), small right subpulmonic effusion, and a new mild patchy left lower lung field opacity suggesting developing atelectasis or pneumonia.

She was diagnosed with acute hydrops of the gallbladder with acute cholecystitis. She was taken to endoscopic retrograde cholangiopancreatography (ERCP) due to possible CBD calculus on MRCP, but the procedure was aborted as the ampulla could not be cannulated and the guidewire could not be passed, likely secondary to the huge gallbladder. Laparoscopic cholecystectomy was performed on the same day after decompressing the gallbladder. The patient’s pain was relieved after the surgery, and her vitals and laboratory results remained stable. She was discharged home two days after cholecystectomy.

## Discussion

The most common condition associated with the gallbladder is gallstone disease. Gallbladder hydrops is a condition that occurs when the cystic duct is blocked for an extended period of time, usually due to an impacted gallstone. Mucocele of the gallbladder is another name for this ailment. When the gallbladder is distended with mucus, water, or clear liquid content instead of bile, it can be diagnosed as gallbladder hydrops [3]. An impacted stone in the gallbladder neck or cystic duct is the most prevalent cause, and it is exactly the same in our patient who had a large stone in her gallbladder neck. Cystic fibrosis, tumors, kinking of the cystic duct, and external compression by inflammatory or neoplastic masses are all less common causes. The bile is slowly resorbed due to a long-standing obstruction, with continuing production of mucoid material from the gallbladder mucosa, resulting in a dilated gallbladder. The wall may be of normal thickness at first, but it might become thin and atrophic over time, and it can even perforate in severe cases [4].

Acute calculous or acalculous cholecystitis can lead to mucoceles. Males and females can acquire gallbladder disease with certain populations having a greater predilection for gallbladder disease. Gallstones affect around 14 million men and six million women in the United States between the ages of 20 and 74. As a person becomes older, the likelihood of contracting the disease rises. The risk factor follows the general rule for four F’s: female, fat (obesity), fertile (multiparity), and forty (age) [5].

Cholelithiasis does not always manifest itself clinically, and it is occasionally discovered during an abdominal ultrasound. From an epidemiological standpoint, roughly 1%-4% of patients may suffer yearly symptoms, with biliary colic (56%) or acute cholecystitis (36%) being the most prevalent presentation [6]. Cholelithiasis is the cause of more than 90% of acute cholecystitis cases. The majority of patients have no symptoms [7]. Symptoms such as pain and inflammation are frequent after an outbreak of acute cholecystitis [8]. Although gallstone disease is more common in the elderly, the rate of acute cholecystitis has decreased since gallstone symptoms are treated with a laparoscopic cholecystectomy [9]. Pain is the immediate effect, but inflammation follows within hours. Inflammation causes the gallbladder to expand, the wall thickens and becomes hyperemic, and a pericholecystic exudate can form [7]. Our patient here has a history of cholelithiasis, and she presented with complaints of right upper quadrant abdominal pain radiating to the back and associated with nausea and vomiting. These symptoms suggested an episode of acute cholecystitis.

Blood tests, USG (abdominal or endoscopic), computed tomography (CT) scan, or hepatobiliary iminodiacetic acid (HIDA) scan are all used to detect this illness [10]. Diagnostic imaging with abdominal USG and MRCP can be performed. Our patient’s RUQ USG showed dilated gallbladder with cholelithiasis, wall thickening, and common bile duct dilatation. Her MRCP demonstrated acute calculous cholecystitis with distal CBD calculus measuring 4 mm and gallbladder hydrops, gallbladder wall thickening, and pericholecystic fluid. Due to possible CBD calculus, the patient was taken for ERCP, but the procedure was aborted as the ampulla could not be cannulated and the guidewire could not be passed, likely secondary to the huge gallbladder. She underwent laparoscopic cholecystectomy on the same day.

Gallbladder hydrops frequently needs hospitalization to reduce gallbladder inflammation. Intravenous hydration, analgesics, and antibiotics are among the acute therapies. The illness is frequently recurring and will eventually necessitate cholecystectomy surgery [10]. Mucocele of the gallbladder is a rare problem that has been treated with laparoscopic cholecystectomy; other surgical options include open or percutaneous cholecystectomy. Laparoscopic cholecystectomy is performed after decompressing the gallbladder. Our patient underwent laparoscopic cholecystectomy after decompressing the gallbladder. The patient’s pain was relieved after the surgery, and her vitals and laboratory results remained stable. She was discharged home two days after cholecystectomy.

## Conclusions

The prevalence of gallstones is a common problem worldwide; therefore, understanding the function and pathologies of the gallbladder is of great clinical significance in routine practice. A hydropic gallbladder can be caused by a blockage of the gallbladder cystic duct, as seen in our patient. Differentiating hydropic gallbladder from other gallbladder conditions is important and should be managed accordingly by acute supportive care and surgical treatment.
